# Anti-vertigo drug prescribing for patients with vestibular symptoms in primary care: a retrospective observational cohort study

**DOI:** 10.3399/BJGPO.2025.0052

**Published:** 2025-12-19

**Authors:** Hà TN Ngo, Otto R Maarsingh, Pauline Slottje, Marco H Blanker, Feikje Groenhof, Jettie Bont, Vincent A van Vugt

**Affiliations:** 1 Amsterdam UMC, location Vrije Universiteit Amsterdam, Department of General Practice, Amsterdam, Netherlands; 2 Amsterdam Public Health Research Institute, Amsterdam, Netherlands; 3 University Medical Centre Groningen, Department of Primary and Long-term Care, Groningen, Netherlands; 4 Amsterdam UMC, location AMC, Department of General Practice, Amsterdam, Netherlands

**Keywords:** vestibular symptoms, dizziness, vertigo, anti-vertigo drugs, inappropriate drug prescribing, general practice

## Abstract

**Background:**

There is limited evidence that anti-vertigo drugs (AVDs) are effective in patients with vestibular symptoms. Still, betahistine is one of the most frequently prescribed off-label drugs. GPs are likely to contribute substantially to these potentially inappropriate prescriptions.

**Aim:**

To evaluate the frequency of (long-term) AVD prescriptions in primary care and characteristics associated with long-term prescriptions.

**Design & setting:**

We conducted a retrospective observational cohort study using anonymised routine primary care data from ≥1.2 million patients registered at 269 general practices throughout the Netherlands, covering the period 2018–2021.

**Method:**

We included adult patients with vestibular symptoms and/or AVD prescriptions. Outcomes were the prevalence and incidence of (long-term) AVD prescriptions. We used a multivariable logistic regression analysis to identify characteristics associated with long-term prescriptions.

**Results:**

Among 66718 patients with vestibular symptoms, 6172 patients (9%) received AVD prescriptions of which 32% were long term. The majority of patients with prescriptions and long-term prescriptions (88% and 77%, respectively) had any other vestibular disorder than Ménière’s disease. Still, Ménière’s disease was associated with long-term prescriptions as well as increasing age. Patients with benign paroxysmal positional vertigo (BPPV) and a symptom diagnosis of lightheadedness were less likely to receive long-term prescriptions, in addition to patients registered at practices in extremely urbanised areas.

**Conclusion:**

AVD prescriptions, including long-term prescriptions, are common among patients with a wide array of vestibular symptoms and disorders, despite limited evidence. Management of vestibular symptoms by GPs can be improved by reducing these potentially inappropriate prescriptions.

## How this fits in

Anti-vertigo drug (AVD) prescriptions, including long-term prescriptions, are common among patients with a wide array of vestibular symptoms and disorders, even though evidence is limited. It remains unclear to what extent GPs contribute to the potentially inappropriate prescribing of AVDs. For that reason, we aimed to evaluate the frequency of (long-term) prescriptions in primary care and characteristics associated with long-term prescriptions. These insights can provide guidance in the reduction and prevention of potentially inappropriate prescriptions.

## Introduction

Vestibular symptoms,^1^ such as dizziness and vertigo, are common in primary care, affecting approximately 1 in 4 adults.^2^ Appropriate management of vestibular symptoms is essential, because they are associated with decreased quality of life, avoidance, isolation, and depression.^3,4^ Most patients with vestibular symptoms are treated in primary care.^5–9^ Notably, GPs consider vestibular symptoms a diagnostic and therapeutic challenge, as patients may experience a variety of sensations that can be caused by a broad spectrum of conditions. A study in primary care showed that nearly 50% of patients aged ≥65 years still experienced dizziness-related impairment after a 10-year follow-up.^10^ This raises the question whether patients with vestibular symptoms receive optimal care. Surveys among Dutch and British GPs showed that only 6%–7% of GPs used vestibular rehabilitation,^11,12^ an effective exercise-based treatment for various vestibular disorders.^13,14^ Meanwhile, it seems to be common practice to prescribe anti-vertigo drugs (AVDs), such as betahistine, despite limited evidence.^15,16^There is some discussion about the evidence for AVDs, especially in patients with Ménière’s disease, and recommendations vary between countries. Betahistine has no Food and Drug Administration (FDA) approval for any vestibular disorder in the US. In the Netherlands and the UK, betahistine was only approved for Ménière’s disease, but is often prescribed for other vestibular disorders.^17–19^ These latter prescriptions can be considered inappropriate, particularly when they are long term.

It remains unclear to what extent GPs contribute to the potentially inappropriate prescribing of AVDs. As more than 80% of patients with vestibular symptoms are treated in primary care,^7–9^ they are likely to contribute substantially. Reduction of inappropriate prescriptions is essential, because they are associated with adverse outcomes and increased healthcare costs.^20,21^ Especially long-term prescriptions should be avoided, owing to accumulating effects.

This study aimed to evaluate the frequency of (long-term) AVD prescriptions in primary care and to identify characteristics associated with long-term prescriptions. These insights can provide guidance in the reduction of (potentially) inappropriate prescriptions.

## Method

### Setting and participants

We performed a retrospective observational cohort study in Dutch primary care, following the Strengthening the Reporting of Observational Studies in Epidemiology (STROBE) statement.^22^ We used anonymised routine primary care data from ≥1.2 million patients registered at 269 general practices. The included general practices participate in the Intercity Collaboration,^23^ which comprises general practice networks in the regions of Amsterdam (Academic Network of General Practice Amsterdam UMC; ANHA), Utrecht (Julius General Practitioners Network; JGPN), Groningen (Academic General Practitioner Network Northern Netherlands; AHON), and Maastricht (Research Network Family Medicine; RNFM). These networks run ongoing longitudinal databases containing pseudonymised data extracted from electronic patient records of all registered patients, except for those who objected to this. The databases can conditionally be used for scientific research that is relevant for (primary) care. Patients are informed about this by their GP.

From March 2018–March 2021, we selected all patients, aged ≥18 years, with vestibular symptoms and/or at least one AVD prescription. We identified patients with vestibular symptoms using International Classification of Primary Care (ICPC) codes^24^ and patients with prescriptions using Anatomical Therapeutic Chemical (ATC) codes.^25^ Supplementary Table 1 details used ICPC and ATC codes.

We collected data on demographic characteristics, diagnosed vestibular disorders, comorbidity, and number of GP consultations for vestibular symptoms. We also gathered data on the prescriptions and characteristics of the general practices. Supplementary Table 2 shows an overview of variables.

### Outcome measures

Outcomes were the prevalence and incidence of (long-term) AVD prescriptions, and patient and/or general practice characteristics associated with long-term prescriptions. We selected these characteristics based on clinical expertise and available literature.^26–29^

We determined the prevalence and incidence of prescriptions only for patients with vestibular ICPC codes. For patients with and without vestibular ICPC codes, we determined the prevalence of long-term prescriptions. We included both groups in the assessment of characteristics associated with long-term prescriptions.

The prevalence was calculated as the total number of patients (with vestibular ICPC codes) who received at least one (long-term) prescription during the 3-year observation period, divided by the average number of patients in the cohort between March 2018 and March 2021. The incidence was calculated as the total number of patients with vestibular ICPC codes and a new prescription divided by the total number of person-years in the cohort per year. Person-years were calculated using the period from registration until deregistration in that specific year. A prescription was considered ‘new’ when a period of at least 6 months had passed between the last date of the prior prescription and the first date of the new prescription. We used data from 2018 to determine whether prescriptions in 2019 were new. Therefore, we only calculated the incidence for 2019 and 2020. Long-term prescriptions were defined as prescriptions for a duration of ≥90 days in a 12-month period. Supplementary Figure 1 shows examples of short-term, long-term, and new prescriptions.

### Statistical analyses

We used descriptive statistics to determine characteristics of patients and general practices, and to calculate the prevalence and incidence of (long-term) prescriptions. We performed univariable logistic regression analyses and a multivariable logistic regression analysis to identify characteristics associated with long-term prescriptions. All variables from the univariable analyses were included in the multivariable analysis. Odds ratios (OR) and 95% confidence intervals (95% CIs) were obtained from the regression analyses. All variables with a *P*-value <0.05 in the multivariable logistic regression analysis were considered statistically significantly associated with long-term prescriptions. We conducted a post-hoc analysis to assess whether the prevalence of prescriptions was affected by the COVID-19 pandemic. We compared the relative change in number of consultations for vestibular symptoms and in number of patients with vestibular ICPC codes who received at least one prescription between 2019 and 2020 (before pandemic) and 2020–2021 (during pandemic). If the number of patients with prescriptions showed a similar change to the number of consultations, the COVID-19 pandemic may have affected the prevalence of AVD prescriptions. We used SPSS Statistics (version 28.0) for the analyses.

## Results

### Prevalence of prescriptions

We included 73 650 patients with vestibular ICPC codes from 269 general practices. For prevalence calculations, we used the average cohort size of 66 718 patients, defined as the mean number of included patients between March 2018 and March 2021. Characteristics of these patients and general practices are shown in Table 1. Additionally, among all patients without vestibular ICPC codes (*n* ≈ 1.2 million), we found 3293 patients who received at least one AVD prescription. The reasons for prescriptions in these patients are unknown.

**Table 1.** Characteristics of patients with vestibular symptoms and their general practices

Patient characteristics^a^ (*n* = 73 650)	
**Age** (median [IQR])	59 years (43–73)
**Gender** (*n* [%])	48 645 female (66)
**Diagnosed vestibular disorder (n ([%)])**	
N17 (Vertigo or dizziness)	31 133 (42)
N17.01 (Rotatory vertigo)	9360 (13)
N17.02 (Lightheadedness)	14 296 (19)
H82 (Vertiginous syndrome)	15 821 (21)
H82.01 (Ménière’s disease)	1650 (2)
H82.02 (Vestibular neuritis)	2503 (3)
H82.03 (BPPV)	13 651 (19)
**Comorbidity** (*n* [%])	
Cardiovascular disease^b^	25 419 (35)
Diabetes^c^	472 (1)
Cerebrovascular disease^d^	5242 (7)
Anxiety disorder^e^	6208 (8)
Depressive disorder^f^	9768 (13)
Polypharmacy^g^	5011 (7)
**General practice characteristics (*n* = 269)**	
**Registered patients** (*n* [%])	
≤5000	176 (65)
>5000	93 (35)
**Employed GPs** (*n* [%])	
1	93 (35)
2	43 (16)
3–4	65 (24)
5–8	48 (18)
>8	19 (7)
Missing	1 (0.4)
**Degree of urbanisation** ^h^ (*n* [%])	
Not or hardly urbanised	57 (21)
Moderately urbanised	27 (10)
Strongly urbanised	58 (22)
Extremely urbanised	124 (46)
Missing	3 (1)

^a^This includes patients with registered vestibular International Classification of Primary Care codes (N17, N17.01, N17.02, H82, H82.01, H82.02, and/or H82.03). ^b^International Classification of Primary Care codes: K86, K87, K83, K84, K75, K76. ^c^International Classification of Primary Care code: T90. ^d^International Classification of Primary Care codes: K89, K90. ^e^International Classification of Primary Care code: P74. ^f^International Classification of Primary Care code: P76.^g^International Classification of Primary Care code: A49.02. ^h^Definition: not or hardly urbanised =<1000 surrounding addresses. Moderately urbanised = 1000–1500 surrounding addresses. Strongly urbanised = 1500–2500 surrounding addresses. Extremely urbanised =>2500 surrounding addresses.

BPPV = benign paroxysmal positional vertigo.

**Figure 1.**
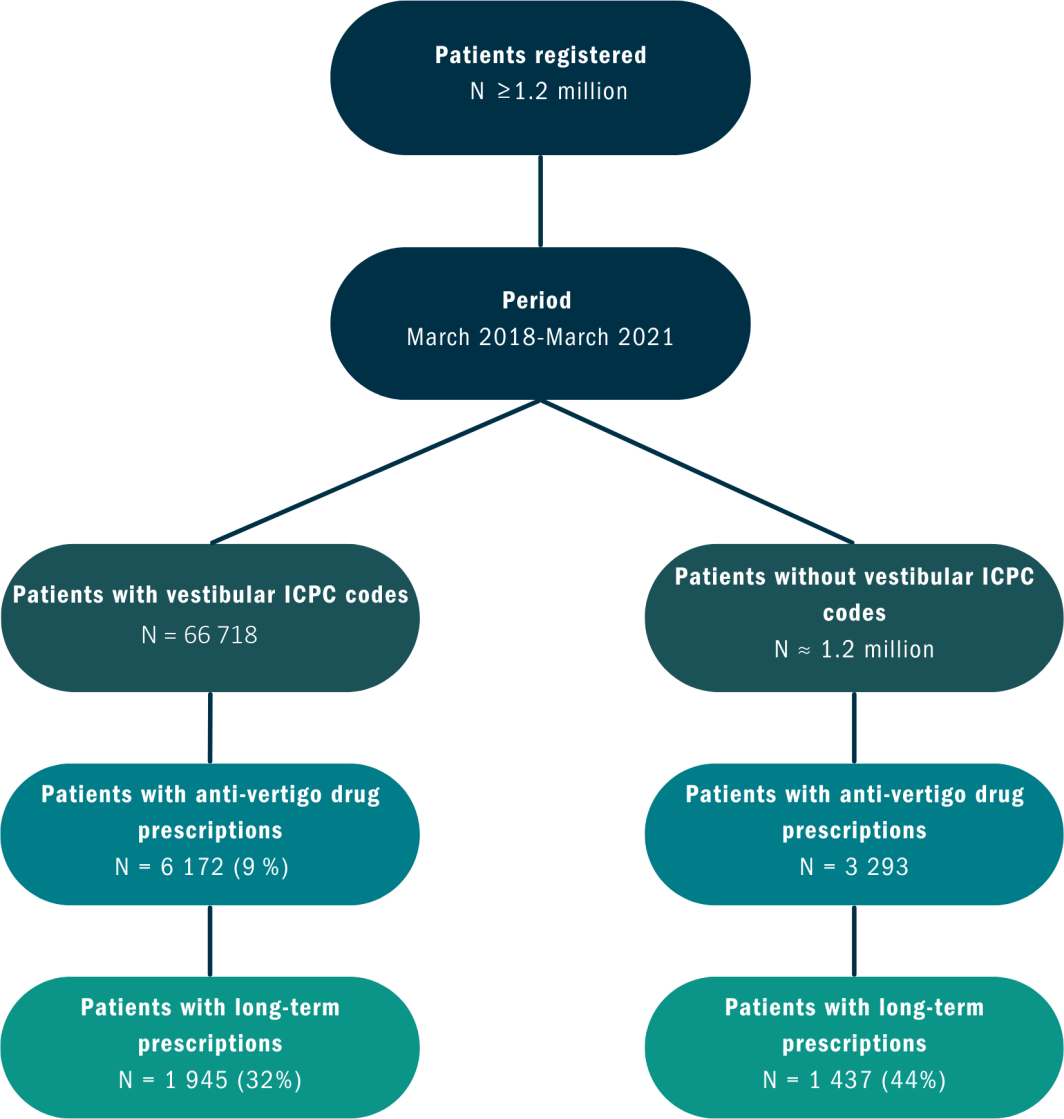
Flowchart showing the patient selection and the number of (long-term) prescriptions. ICPC = International Classification of Primary Care

Among all patients with vestibular ICPC codes, 6172 patients received at least one AVD prescription in the observation period. Using the average cohort size of 66 718 patients, the 3-year period prevalence was 9%. Figure 1 shows a flowchart of the patient selection and prescriptions.

Among 6172 patients with vestibular ICPC codes and AVD prescriptions, 750 patients (12%) had an ICPC code for Ménière’s disease. The remaining 5422 patients (88%) had ICPC codes for unspecified vestibular disorders and/or codes for vestibular disorders other than Ménière’s disease. Betahistine was prescribed in 4674 of 6172 patients (76%), cinnarizine in 1849 patients (30%), and flunarizine in 43 patients (0.7%).

**Figure 2.**
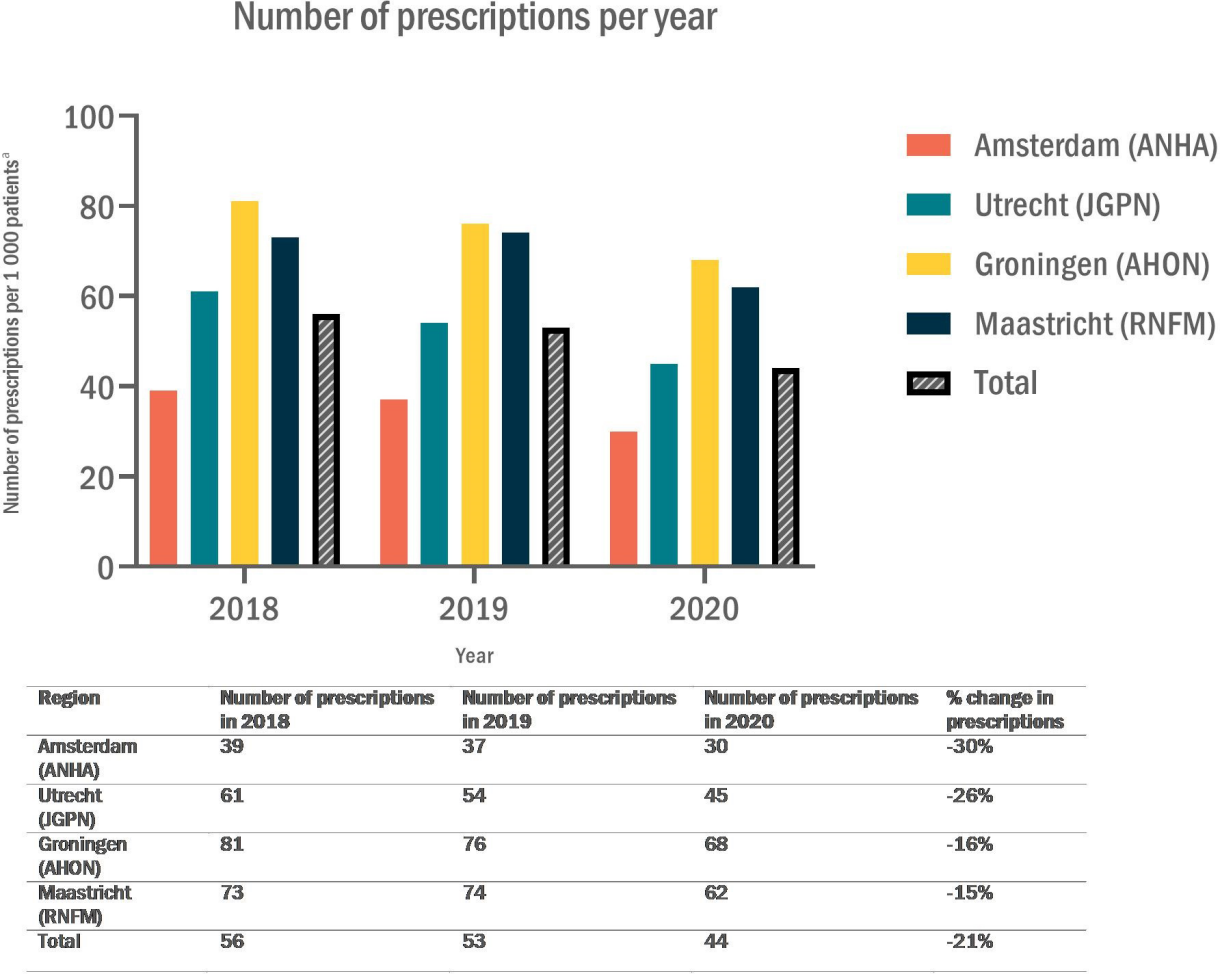
Number of patients with vestibular International Classification of Primary Care codes (N17, N17.01, N17.02, H82, H82.01, H82.02, H82.03) who received at least one anti-vertigo drug prescription.^a^ per 1000 patients per year and the relative change in this number in 2020 versus 2018, per region in the Netherlands and for the total cohort (all practices combined). Anatomical Therapeutic Chemical codes: N07CA01, N07CA02, N07CA03, N07CA52. AHON = Academic General Practitioner Network Northern Netherlands. ANHA = Academic Network of General Practice Amsterdam UMC. JGPN = Julius General Practitioners’ Network. RNFM = Research Network Family Medicine

During the observation period, the prevalence of prescriptions for patients with vestibular ICPC codes decreased from 56 prescriptions per 1000 patients per year to 44 prescriptions per 1000 patients per year (-21%). Figure 2 shows the prevalence of prescriptions per year. We also observed a decrease of 32% in the number of patients without vestibular ICPC codes who received at least one prescription in 2020 versus 2018 (Supplementary Figure 2).

### Secondary outcomes

#### Incidence of prescriptions

The incidence of AVD prescriptions in patients with vestibular ICPC codes was 41 per 1000 person-years in 2019 and 33 per 1000 person-years in 2020 (-20%). Supplementary Figure 3 shows the incidence per region.

#### Prevalence of long-term prescriptions

Among 6172 patients with vestibular ICPC codes and AVD prescriptions, 1945 patients (32%) received long-term prescriptions. Among these 1945 patients, 452 patients (23%) had an ICPC code for Ménière’s disease. The remaining 1493 patients (77%) had any other vestibular ICPC code. Among 3293 patients who received prescriptions without vestibular ICPC codes, 1437 patients (44%) received long-term prescriptions. In total, 36% of all patients with vestibular ICPC codes and without vestibular ICPC codes with prescriptions received long-term prescriptions.

#### Characteristics associated with long-term prescriptions

Table 2 shows the results of the univariable and multivariable logistic regression analyses. The odds of receiving long-term prescriptions increased with age (odds ratio [OR] 1.04 [95% confidence interval {CI} = 1.03 to 1.04]). Patients with Ménière’s disease were also more likely to receive long-term prescriptions (OR 4.15 [95% CI = 3.42 to 5.05]). In contrast, long-term prescriptions were less likely in patients with benign paroxysmal positional vertigo (BPPV) (OR 0.68 [95% CI = 0.57 to 0.83]) and the symptom diagnosis lightheadedness (OR 0.54 [95% CI = 0.39 to 0.74]). Furthermore, we found that long-term prescriptions were less common in patients registered at general practices in extremely urbanised areas (OR 0.82 [95% CI = 0.72 to 0.93]).

**Table 2.** Characteristics associated with long-term anti-vertigo drug prescriptions (that is, prescriptions for a total duration of 90 days or more in 12 months’ time)

**Characteristic**	**Long-term prescription** **(≥90 days within 12 months)** **(*n* = 3382)**	**No long-term prescription (<90 days within 12 months)** **(*n* = 6079)**	**Univariable analysis (OR [95% CI])**	**Multivariable analysis (OR [95%CI])**
**Age** (median [IQR])	73 (62–82)	62 (49–75)	1.04 (1.04 to 1.04)^a^	1.04 (1.03 to 1.04)^a^
**Sex, female** (%)	71	72	0.99 (0.91 to 1.09)	1.03 (0.93 to 1.14)
**Vestibular symptom or disorder** (%)				
N17 (Vertigo or dizziness)	15	13	1.16 (1.02 to 1.31)^a^	1.06 (0.93 to 1.21)
N17.01 (Rotatory vertigo)	4	5	0.86 (0.70 to 1.05)	0.87 (0.70 to 1.08)
N17.02 (Lightheadedness)	2	3	0.62 (0.46 to 0.83)^a^	0.54 (0.39 to 0.74)^a^
H82 (Vertiginous syndrome)	9	9	1.05 (0.91 to 1.22)	0.97 (0.83 to 1.14)
H82.01 (Ménière’s disease)	11	3	4.44 (3.68 to 5.34)^a^	4.15 (3.42 to 5.05)^a^
H82.02 (Vestibular neuritis)	1	2	0.73 (0.50 to 1.05)	0.82 (0.56 to 1.21)
H82.03 (BPPV)	5	8	0.62 (0.52 to 0.75)^a^	0.68 (0.57 to 0.83)^a^
**Comorbidity** (%)				
K86 (Hypertension, uncomplicated)	36	26	1.56 (1.43 to 1.71)^a^	1.07 (0.96 to 1.18)
K87 (Hypertension, complicated)	7	5	1.56 (1.29 to 1.87)^a^	1.02 (0.84 to 1.25)
K83 (Heart valve disease)	2	1	1.54 (1.09 to 2.16)^a^	0.96 (0.67 to 1.38)
K84 (Heart disease, other)	2	1	2.11 (1.50 to 2.96)^a^	1.35 (0.94 to 1.95)
K75 (Acute myocardial infarction)	6	4	1.70 (1.39 to 2.08)^a^	1.12 (0.90 to 1.39)
K76 (Ischaemic heart disease without angina)	2	1	1.76 (1.26 to 2.46)^a^	1.16 (0.82 to 1.65)
K89 (Transient cerebral ischaemia)	6	4	1.57 (1.29 to 1.91)^a^	0.97 (0.79 to 1.19)
K90 (Stroke or cerebrovascular accident)	4	2	1.55 (1.22 to 1.97)^a^	1.00 (0.77 to 1.29)
T90 (Diabetes)	1	1	1.35 (0.79 to 2.30)	1.22 (0.70 to 2.13)
P74 (Anxiety disorder or anxiety state)	5	6	0.86 (0.71 to 1.03)	1.05 (0.86 to 1.29)
P76 (Depressive disorder)	10	11	0.91 (0.79 to 1.04)	1.03 (0.89 to 1.20)
A49.2 (Polypharmacy)	7	4	1.79 (1.49 to 2.14)^a^	1.06 (0.87 to 1.29)
**Registered patients** (%)				
>5000 (in comparison with≤5000)	55%	53%	1.11 (1.02 to 1.21)^a^	1.12 (0.99 to 1.26)
**Employed GPs** (%)				
1^b^	25	27	1.0	1.0
2	12	11	1.14 (0.98 to 1.32)	1.15 (0.98 to 1.35)
3–4	28	27	1.11 (0.99 to 1.24)	1.06 (0.93 to 1.20)
5–8	23	23	1.06 (0.94 to 1.19)	1.11 (0.95 to 1.30)
>8	12	12	1.10 (0.95 to 1.27)	1.04 (0.86 to 1.26)
**Degree of urbanisation** (%)^c^				
Not or hardly urbanised^b^	23	21	1.0	1.0
Moderately urbanised	17	13	1.14 (0.99 to 1.32)	1.10 (0.94 to 1.28)
Strongly urbanised	29	29	0.90 (0.80 to 1.01)	0.95 (0.83 to 1.08)
Extremely urbanised	30	36	0.75 (0.66 to 0.84)^a^	0.82 (0.72 to 0.93)^a^

^a^Significant association, *P*<0.05. ^b^Reference category. ^c^Definition: not/hardly urbanised = <1000 surrounding addresses. Moderately urbanised =1000–1500 surrounding addresses. Strongly urbanised = 1500–2500 surrounding addresses. Extremely urbanised = >2500 surrounding addresses. BPPV = benign paroxysmal positional vertigo. CI = confidence interval*. *OR = odds ratio

### Post-hoc analysis

We found 1028 consultations for vestibular symptoms per 1000 patients per year in 2019–2020 (before the COVID-19 pandemic) and 1019 consultations per 1000 patients per year in 2020–2021 (during the pandemic): a decrease of 0.9%. The number of AVD prescriptions decreased from 53 per 1000 patients with vestibular ICPC codes per year in 2019–2020 to 44 per 1000 patients per year in 2020–2021 (-17%).

## Discussion

### Summary

We assessed the frequency of (long-term) AVD prescriptions in primary care and characteristics associated with long-term prescriptions.

Among patients with vestibular symptoms, 9% received AVD prescriptions, and 32% of these were long term. The majority of patients with prescriptions had ICPC codes for unspecified vestibular disorders and/or codes for vestibular disorders other than Ménière’s disease. The same is true for patients with long-term prescriptions. Ménière’s disease was associated with long-term prescriptions as well as increasing age. Patients with BPPV and lightheadedness were less likely to receive long-term prescriptions, in addition to patients registered at practices in extremely urbanised areas. We observed a decrease of 21% in prevalence of prescriptions during the 3-year observation period.

### Strengths and limitations

The first strength of this study is the representativeness, supported by several factors. In the Netherlands, nearly all non-institutionalised citizens are registered at a general practice, where GPs act as gatekeepers. Additionally, this study included diverse general practices, varying in patient population and practice size. Furthermore, by using data over a 3-year observation period from more than 1.2 million patients registered at 269 practices throughout the Netherlands, we were able to include data from a substantial part of the Dutch population (estimated at 18 million people).^30^ Even though our results are representative for the Dutch population, it might be difficult to apply the results internationally, as healthcare systems (and therefore the GP’s role) may differ as well as prescription patterns. Still, a recent study using national health insurance data in France showed that betahistine prescriptions exceeded the prevalence of Ménière’s disease, while it is not recommended as a first-line treatment for this disease.^19^ The study does not report how many prescriptions were made in primary care, but it does suggest that the issue of overprescribing is prevalent in other countries. Our second strength is the minimal risk of missing data regarding prescriptions by GPs, taking aforementioned into account and the fact that betahistine is not available as an over-the-counter drug. Lastly, pooling of the data allowed us to accurately evaluate characteristics associated with long-term prescriptions.

Limitations of this study are related to the use of routine data. First, incomplete or inaccurate registration of ICPC codes may have reduced the quality of the data. For instance, a part of AVDs were prescribed in patients without a vestibular ICPC code. Second, ICPC codes that describe symptoms, rather than disorders, may represent undiagnosed (vestibular) disorders, such as Ménière’s disease. Still, it is potentially inappropriate to prescribe AVDs in most vestibular disorders and/or in case of an unclear diagnosis, especially when prescriptions are long term. Third, we were unable to differentiate between prescriptions that were started in primary care versus in secondary care. It would be interesting to combine data from primary and secondary care to evaluate prescription patterns more accurately. Lastly, we were restricted in evaluating which characteristics were associated with long-term prescriptions and how these characteristics were defined. A potentially relevant characteristic such as level of education of patients could not be ascertained at this time. Continuous improvement of primary care databases and linkage with other reliable data resources may remedy aforementioned problems in the future.^11,12^

We did not include data on non-pharmacological treatments, such as vestibular rehabilitation, as it was out of the scope of this study. Prior studies suggest that this evidence-based treatment is underused in primary care^11,12^ and it would be useful to evaluate this more thoroughly. We will include these data in the evaluation of our current nationwide implementation study, in which our aim is to improve the uptake of vestibular rehabilitation by implementing an online version of it.^31^

### Comparison with existing literature

Two studies in Dutch primary care reported prevalences of AVD prescriptions in older patients. One study showed that GPs prescribed AVDs in 9.2% of patients aged ≥65 years with non-vestibular dizziness during their first consultation.^26^ The other study showed that 5.1% of patients aged ≥65 years who visited their GP for dizziness received a prescription.^9^ These prevalences are lower than expected, as the prevalence of vestibular symptoms increases with age and our study showed that older age is associated with long-term prescriptions. The discrepancies may be explained by different inclusion criteria. The first study only included patients with non-vestibular dizziness, whereas we included patients regardless of the cause. The second study did not include patients who received prescriptions without a vestibular ICPC code, as we did in our study. AVD prescriptions have been evaluated in other countries too: in German primary care, 6.6% of all patients with dizziness received AVD prescriptions,^32^ while a study in the US found that primary care physicians prescribed medication in 61% of patients with dizziness who sought care. Meclizine was most prescribed, but it is unclear what other drugs were given.^8^ Aforementioned studies are, however, from decades ago and are therefore not representative for the current management of vestibular symptoms.

AVDs were frequently prescribed in our study, but we did observe a decrease of 21% in the prevalence of prescriptions during the observation period. ^33^ ^34^The decrease observed in our study between 2018 and 2020 is consistent with national trends. Data from the GIP database — a database hosted by the Dutch National Health Care Institute that provides annual numbers of unique users per ATC code — show that the number of users of ATC-subgroup N07C continued to decline nationally, from 66,528 users in 2020 to 39,210 users in 2024 (–41%), indicating an even steeper decrease in the years following our study period.

No prior studies evaluated characteristics associated with long-term AVD prescriptions. We found that the prevalence of long-term prescriptions increased with age. Inappropriate prescribing, including overprescribing, is a common issue among older patients.^35^ GPs may experience difficulties with deprescribing, owing to various factors,^36^ which may lead to more long-term prescriptions in older patients.

Additionally, long-term prescriptions were more likely in patients with Ménière’s disease. This is in line with our expectations, as historically betahistine was registered as a treatment for this condition.^37^ Cochrane reviews have shown that there is limited evidence for its effectiveness.^16,38^ Still, guidelines on the management of Ménière’s disease vary between countries; for example, some recommend AVDs, while others, including the Dutch guideline, have advised against this for decades.^39^ In spite of that, Dutch GPs seem to persist in prescribing AVDs, not only for patients with Ménière’s disease, but also for other vestibular disorders as well. These prescriptions are potentially inappropriate, especially when prescribed long term. In addition to potential adverse effects on an individual level, stopping betahistine prescriptions could substantially decrease healthcare costs; for example, British studies show that more than 4 000 000 GBP per year could be saved.^18,37^

Long-term prescriptions were less prevalent in patients with BPPV and lightheadedness, which was anticipated. BPPV is a short-lasting and self-limiting disorder, and repositioning maneuvers have proven to be effective.^40^ Lightheadedness is often seen as a sign of cardiovascular disease, which explains why GPs are less likely to prescribe AVDs in these patients.

Lastly, we found that long-term prescriptions were less likely in practices located in extremely urbanised areas. This is contradictory to Dutch studies on the inappropriate prescribing of antibiotics and opioids.^41,42^ Overrepresentation of practices in extremely urbanised areas in our dataset (46% compared with 26% in the Netherlands)^43^ may have led to an underestimation of long-term prescriptions. However, this supports the importance of reducing AVD prescriptions even more.

### Implications for research and practice

AVD prescriptions, including long-term prescriptions, are common among patients with vestibular symptoms in primary care, despite limited evidence. The majority of patients with AVD prescriptions had unspecified vestibular diagnoses or vestibular diagnoses for which other evidence-based treatments are available. Although we observed a decrease in prescriptions over the years, management of vestibular symptoms by GPs can still be improved by further reducing these potentially inappropriate prescriptions. Our current qualitative study with GPs and patients will explore barriers and facilitators to prescribing and using AVDs, which may guide the deprescribing of AVDs.

## Data

The dataset relied on in this article is available from the corresponding author on reasonable request.
